# Job Coaching and Smartphone-Based Cognitive Behavioral Techniques for Returning to Work and Coping With Unemployment (DRIVEN): Randomized Controlled Trial

**DOI:** 10.2196/90873

**Published:** 2026-10-20

**Authors:** James W Griffith, Allison M Letkiewicz, Mystie L Saturday, Andrea K Graham, Keith Alperin, Elizabeth C Danielson, Alexandra Levit, Stewart A Shankman

**Affiliations:** 1 Department of Obstetrics and Gynecology, University of Chicago, 5841 South Maryland Avenue, Chicago, IL, 60637, United States, 1 773 702 1971; 2 Department of Psychiatry and Behavioral Neuroscience, University of Chicago, Chicago, IL, United States; 3 University of Chicago Comprehensive Cancer Center, Chicago, IL, United States; 4 Department of Psychiatry and Behavioral Sciences, Stephen M. Stahl Center for Psychiatric Neuroscience, Northwestern University, Chicago, IL, United States; 5 Department of Psychology, University of Chicago, Chicago, IL, United States; 6 Department of Medical Social Sciences, Northwestern University, Chicago, IL, United States; 7 Helium Foot Software, Inc, Chicago, IL, United States; 8 Department of Surgery, Surgical Implementation & Health Services Research, Biological Sciences Division, University of Chicago, Chicago, IL, United States; 9 Inspiration at Work, Chicago, IL, United States

**Keywords:** unemployment, digital health intervention, depression, cognitive behavioral therapy, randomized controlled trial

## Abstract

**Background:**

Unemployment has economic consequences and is associated with emotional distress and impairment. Joblessness is often associated with depression, leading to a vicious cycle of reduced drive to seek work, deepening financial stress, and worsening depression. Few scalable interventions exist that integrate job-search strategies with cognitive behavioral techniques.

**Objective:**

The study aimed to determine the efficacy of a Distress-Informed Return-to-Work Intervention (DRIVEN). The primary hypothesis was that DRIVEN would be more efficacious than the control condition in improving job-search behavior. Secondary analyses examined job attainment, depression, and positive affect.

**Methods:**

A randomized controlled trial was conducted from August 2023 to September 2024. Assessments were at baseline, weeks 1 through 6, and 8 and 16 weeks postintervention. Participants were a nationwide sample of unemployed job seekers aged 18 years or older who had never earned more than US $100,000 in a year. Participants received either (1) DRIVEN, which included 6 weeks of cognitive behavioral exercises in a mobile app combined with three 1-hour coaching sessions over videoconference or (2) emailed articles containing job-search advice. Primary outcome measures were subscales of a modified Job Search Behavior Scale (preparatory and active job search). Secondary outcomes included job attainment and mental health outcomes.

**Results:**

Participants were a nationwide sample (N=126; mean age 33.6, SD 6.6 y), including 62 (49.2%) females. During the acute treatment phase, mixed-effects linear models showed that for DRIVEN, preparatory job-search behavior increased by an estimated 0.053 (95% CI 0.031-0.076) units per day from baseline, which was a larger effect than in the control condition (0.011, 95% CI −0.011 to 0.033 units per day; *P*=.008). Active job-search behavior increased by 0.061 (95% CI 0.038-0.084) units per day from baseline, which was significantly larger than in the control group (point estimate: 0.025, 95% CI 0.001-0.047; *P*=.03). Depression decreased more in the DRIVEN group vs control (DRIVEN point estimate=−0.214, 95% CI −0.255 to −0.172 vs control group point estimate=−0.122, 95% CI −0.163 to −0.081; *P*=.002), and participants were more likely to get a job in the DRIVEN group (68.3% vs 50.8%; *P*=.046). Self-report outcomes did not significantly differ during the follow-up periods.

**Conclusions:**

DRIVEN was superior to emailed job-search advice for the primary outcome (job-search behavior) and secondary outcomes (job attainment, depression, and positive affect). This intervention may be beneficial for distressed unemployed individuals seeking work.

## Introduction

Employment is a social determinant of health that can affect an individual’s economic status, social connection, and access to health care, especially in the United States, where health insurance is often obtained through an individual’s employer [1-3]. As of May 2026, the US Department of Labor reported that approximately 4.3% of the population (over 7 million individuals) were unemployed [4], which not only creates costs to the government to cover unemployment insurance but also leaves these individuals without income, social connections, and a sense of meaning and purpose [2]. As such, it is unsurprising that unemployment has been associated with both symptoms of depression and major depressive disorder [5]. Joblessness also can lead to a vicious cycle of reduced drive to seek work, deepening financial stress, and worsening depression [6-8]. Thus, there is an urgent need for scalable programs that can both treat emotional distress—such as depression and anxiety—as well as teach effective job-search strategies. In fact, previous meta-analyses of job-search interventions have found they can reduce depression, but few studies have assessed mental health outcomes more broadly [9]. For these reasons, we created a smartphone app–delivered intervention for job seekers (Distress-Informed Return-to-Work Intervention [DRIVEN]) that (1) integrates job-search strategies with cognitive and behavioral techniques to address emotional distress and diminished motivation and (2) includes brief videoconference meetings with a job coach to support motivation, review intervention techniques, and discuss job-search strategies. In this way, DRIVEN combines the strong evidence for cognitive behavioral therapy (CBT) for depression [10] with practical support and coaching specific for a job search.

This research builds upon existing evidence that cognitive behavioral skills may aid job seekers. For example, in a sample of 75 job seekers, cognitive behavioral skills predicted job-search self-efficacy, even after covarying for depression [11]. In addition, Della-Posta and Drummond [12] found unemployed job seekers (n=39) experienced less distress and were more likely to find work when receiving a combination of cognitive behavioral techniques and job-search support vs standard job-search support alone. In another study, unemployed individuals completed either a 7-week CBT (n=94) or control program (n=89) that both emphasized social support. After the program, the CBT group reported better mental health outcomes and more participants found full-time employment [13]. The purpose of this research was to build upon previous studies to determine whether DRIVEN—which was designed to be brief, mostly self-guided, and smartphone-based—could help unemployed job seekers attain jobs and improve mental health.

Our primary hypothesis was that the DRIVEN intervention would lead to more job-search behavior at a postintervention assessment point and follow-ups at 8 and 16 weeks, relative to a control condition designed to reflect usual job-search behavior (ie, weekly emails containing articles and recommendations for job seekers). Secondary outcome measures included self-report measures of depression, positive affect, and job attainment.

## Methods

### Overview

This study was a 2-arm, parallel-assignment randomized controlled trial (RCT). Job-search behaviors and other outcomes were captured at baseline, during each of the 6 weeks of the intervention, and again at the 8- and 16-week postintervention follow-ups. Additional details about the study design, study protocol, and statistical analysis plan can be found in our protocol study [14]. The study was funded by the National Institute of Mental Health; the sponsor had no role in the design, conduct, analysis, or reporting of the trial. Throughout the study, an advisory board was maintained, comprising community members and experts in human resources, business, clinical psychology, and community engagement. A Data Safety Monitoring Board comprising a clinical psychologist with expertise in digital interventions and 2 statisticians was maintained; it met midway through the trial and approved continuation based on its review of data and procedures. The first participant was enrolled on August 7, 2023. The study was retrospectively registered with ClinicalTrials.gov on August 30, 2023, and the registration was publicly visible on September 7, 2023.

### Ethical Considerations

Data were stored in REDCap, which allowed data to be exported for analysis without identifying information, such as names and addresses [15]. Access to the REDCap database was available only to approved study team members, and data files used for analysis were kept on secure computers. Participants received compensation for the completion of study assessments of up to US $350 (US $50 for the baseline assessment; US $20 for each of the first 3 weeks of questionnaires; US $30, US $40, and US $50 for the fourth, fifth, and sixth weeks of questionnaires, respectively; and US $60 for each of the 2 follow-ups). Compensation was not provided for time spent on the intervention procedures in either of the 2 groups. Advarra served as the Ethics Review Board (Pro00062697) and approved the study; all participants provided written informed consent.

### Randomization

A randomization list, stratified by gender, was created by JWG using the *blockrand* package for R (R Foundation for Statistical Computing) [16], which used randomly varying block sizes and then equally allocated participants to groups within the block (ie, the study used a 1:1 allocation ratio). Block sizes were 2, 4, 6, and 8. A seed was set for reproducibility. The randomization list was uploaded by a study coordinator to REDCap [15] and then used for sequential participants, within gender. After randomization, participants were onboarded by study coordinators (who had access to the randomization in REDCap) to their respective arms of the study. Participants were told in the consent form “You will be randomized to one of two approaches that are designed to give you the tools to help you (a) cope with the stress of being unemployed and having to search for work and (b) navigate your job search most effectively.” Thus, participants did not receive information about which group was the DRIVEN group vs the control group.

### Participants

#### Overview

Participants were English-speaking, unemployed adult job seekers aged 18 years or older who had never received an annual salary of more than US $100,000; detailed inclusion and exclusion criteria are shown in Table S1 in Multimedia Appendix 1.

#### Setting and Eligibility

Using online advertisements on Craigslist, 126 participants were recruited from 7 US metropolitan areas, selected for their geographic diversity (New York, Los Angeles, Chicago, Houston, Jacksonville, Philadelphia, and Phoenix). The sample size (N=126) was determined by our a priori power analyses; the study protocol specified a maximum of 150 participants to account for the possibility of overrecruitment. Recruitment took place from August 2023 to September 2024. Eligible participants were 18 to 65 years of age, currently unemployed (defined as working <8 h/wk), actively searching for work, previously employed full-time (≥35 h/wk) for at least 1 year prior to unemployment, had a highest individual salary of US $100,000 or less, owned an iPhone (Apple Inc) or Android (Google LLC) smartphone, were able to provide informed consent, had a minimum depression score according to the Patient-Reported Outcomes Measurement Information System (PROMIS) Depression 8a scale (T-score of 45, which is within 0.5 SD of the mean of the US general population but above the floor of the questionnaire), and were able to communicate in English (verbal and written). Exclusion criteria included current (at the time of enrollment) moderate or severe alcohol/substance use disorders, suicidal planning and/or active suicidal ideation, current manic or psychotic symptoms, or being a full-time/part-time student. Research coordinators were available to both groups in case adverse events needed to be reported.

#### Sample Size Justification

Using the pwr.r.test() function in R [17], we estimated that 100 participants (50 per group) would yield 93% power for a between-group delta of 0.7 standardized units, assuming a 2-sided test; the sample size was inflated to 126 to account for a possible dropout rate of 20%.

### Intervention Procedures

Table 1 summarizes the content of the DRIVEN intervention as well as that of the control group.

**Table 1.** Weekly comparison of intervention components of DRIVEN^a^ vs the control group.

Week	Intervention focus (DRIVEN mobile app)	Coaching session focus (DRIVEN participants only)	Control group
1	Setting expectations Connections among thoughts, feelings, and behavior Thinking flexibly	—^b^	Best websites for job searching How to apply for a job online (including resume samples and salary calculator)
2	Self-monitoring of symptoms and stress Thinking flexibly and taking action as coping tools	Welcome, rapport-building, and recap of a flexible thinking exercise and content from week 1	Organizing the job search Skills self-assessment
3	Building skills Understanding employers’ needs Finding a job that is a good fit	—	Resume writing with links to examples and templates Hard vs soft skills Resume keywords and getting noticed
4	Understanding internet-based applications Cover letters and resumes	Recognizing stress Implementing flexible thinking and taking action to address it Employer-preferred skills Identifying ideal jobs	More detail on a resume that stands out Cover letters
5	Networking Interviewing	—	Preparing for a job interview Common interview questions and answers Negotiation
6	Recap of the program Putting things into practice (after the intervention is over)	Making use of social connections (which might lead to opportunities) Review of the program and the participant’s progress	Networking Asking people to serve as a reference

a DRIVEN: Distress-Informed Return-to-Work Intervention.

b Not applicable.

#### DRIVEN

The DRIVEN intervention was designed to combine skills derived from CBT*—thinking flexibly* (ie, cognitive reappraisal) and *taking action* (ie, behavioral activation)—applied to unemployment-related distress and job searching (eg, coping with rejection after a job interview). Software development was led by KA, who met with the research team to outline the mobile app, built necessary features for DRIVEN (eg, sequential exercises, videos, and a resource list), and integrated changes based on pilot testing. The intervention was 6 weeks long, with weekly, self-paced activities delivered through participants’ iPhones or Android smartphones. Activities included instructional and/or motivational videos, activities related to self-monitoring (eg, a weekly mood self-assessment), and self-guided cognitive reappraisal activities.

A coaching manual was created by the authors, and each participant received three 1-hour sessions with a job coach via videoconference. Coaching was oriented toward providing individualized guidance to the participants on topics including job searching, networking, resume review, cover letters, reviewing homework during the intervention, and interview preparation.

The app’s information was delivered in a fixed sequence so that all participants had a consistent experience. Screenshots of representative activities are presented in Multimedia Appendix 1.

#### Control Group

The control group received weekly emails with information about job searching. It was designed to be an “active” control group with helpful articles that a person might find useful, rather than an “inert” control group. Table 1 summarizes the contents of the emails, which were intended to reinforce “job seeking as usual” by providing helpful and supportive advice, without using principles of CBT.

### Study Measures

#### Job-Search Behaviors

Job-search behaviors were measured using a variant of the Job Search Behavior Scale proposed by Blau [18]. Adaptations to the questionnaire used updated wording to reflect modern job-search practices (referencing “job postings” rather than “classified ads in a newspaper”). The questionnaire assessed the number of times participants engaged in 10 different job-search activities and divided into 2 subscales: preparatory job-search behaviors (5 items; eg, preparing/revising resume and speaking with previous employers about possible job leads) and active job-search behaviors (5 items; eg, going on a job interview and sending out applications). Items are scored on a 1 to 5 scale from “0 times” to “6 or more times,” and subscales are scored by summing the items. During the intervention period (baseline through week 6), participants reported weekly on job-search behaviors. At the 2 follow-up periods (8 and 16 weeks postintervention), participants were asked to report on job-search behaviors over the past 2 months.

#### PROMIS Emotional Distress-Depression—Short Form 8a

This PROMIS Depression 8a short form contains 8 items rated using a 5-option rating scale from “Never” to “Always,” rated over the past 7 days. The PROMIS depression item bank showed a high degree of convergent validity with other depression questionnaires (Pearson correlation coefficient *r* of 0.86 for the Center for Epidemiological Studies Depression scale and 0.81 for the Patient Health Questionnaire-9) and a reliability coefficient of 0.92 [19]. The PROMIS depression scales are also responsive to changes in clinical status [20] and have been extensively used in RCTs.

#### PROMIS Positive Affect—Short Form 15a

This 15-item questionnaire asks about how much positive emotion has been experienced over the past 7 days, using a 5-item rating scale ranging from “Not at all” to “Very much.” These items were derived from an item bank developed for the National Institutes of Health Toolbox, which has a high level of reliability [21].

#### Credibility and Expectations

At the start of the trial, participants completed a psychometrically valid questionnaire on treatment credibility and expectations for behavioral interventions [22]. Factor analysis of this questionnaire showed 2 latent factors—credibility and expectations—with 3 items each (sample item: “How confident would you be in recommending this treatment to a friend?”).

### Statistical Methods

R statistical software (version 4.5.3) was used for data analyses [23]. A mixed-model regression analysis, implemented in the *nlme* package of R [24], was used to compare job outcomes in the intervention group vs the control group. The baseline value was included in the model as a covariate, time was included in the model as a continuous variable (days since baseline), and a subject-specific random intercept was included in the model. A group × time interaction effect tested whether the intervention group influenced the trajectory of outcomes over time, and quadratic components were explored in the modeling to test for possible nonlinear changes over time. Separate models were fitted for follow-up data. Mixed model regression was chosen for the analysis, in part, because of the ability to include cases in the analyses even in the context of missing data, assuming that the data were missing at random. The proportion of people who obtained a job was compared using a chi-square test; survival analyses assessed whether time-to-job-finding was different between groups (survival analyses are presented in the Supplemental Results in Multimedia Appendix 1). All randomized participants were included for intent-to-treat analyses. A type I error rate of 0.05 was used for 2 primary outcomes without adjustment for familywise error rate.

## Results

### Sample Characteristics and Engagement in Intervention

Descriptive statistics are presented in Table 2, which show that participants were roughly evenly divided between men and women and were diverse with respect to race and ethnicity. Table S2 in Multimedia Appendix 1 presents participants’ engagement with the mobile app based on 3 activity types—cognitive behavioral exercises designed to help cope with unemployment-related distress, mood self-assessments, and instructional and/or motivational videos. Engagement was high (84.4%‐86.7%) among participants randomized to the DRIVEN condition. A 2025 CONSORT (Consolidated Standards of Reporting Trials) diagram is presented in Figure 1 [25], which shows the flow of participants through the study from screening to completion (Checklist 1). Table 3 shows the marginal values of the means, SEs, and between-group standardized mean differences (and corresponding 95% CIs) for preparatory job-search behavior, as well as for active job-search behavior, depression, and positive affect.

**Table 2.** Demographic characteristics of participants in the randomized controlled trial.

Characteristics	Control (n=63)	Driven (n=63)	Overall (N=126)
Age (y), mean (SD)	34.2 (7.0)	33.0 (6.3)	33.6 (6.6)
Gender, n (%)			
Women	30 (47.6)	32 (50.8)	62 (49.2)
Men	33 (52.4)	31 (49.2)	64 (50.8)
Nonbinary	0 (0)	0 (0)	0 (0)
Something else	0 (0)	0 (0)	0 (0)
Prefer not to respond	0 (0)	0 (0)	0 (0)
Ethnicity, n (%)			
Hispanic/Latino	14 (22.2)	13 (20.6)	27 (21.4)
Not Hispanic/Latino	48 (76.2)	50 (79.4)	98 (77.8)
Unknown	1 (1.6)	0 (0)	1 (0.8)
Prefer not to respond	0 (0)	0 (0)	0 (0)
Race, n (%)			
American Indian/Alaskan Native	1 (1.6)	1 (1.6)	2 (1.6)
Asian	1 (1.6)	1 (1.6)	2 (1.6)
Native Hawaiian or other Pacific Islander	0 (0)	0 (0)	0 (0)
Middle Eastern	0 (0)	0 (0)	0 (0)
India subcontinent	0 (0)	0 (0)	0 (0)
Black or African American	47 (74.6)	51 (81.0)	98 (77.8)
White	11 (17.5)	9 (14.3)	20 (15.9)
More than one race	2 (3.2)	1 (1.6)	3 (2.4)
Prefer not to respond	0 (0)	0 (0)	0 (0)
Unknown	1 (1.6)	0 (0)	1 (0.8)
How long since working full time?, n (%)			
<2 weeks	0 (0)	0 (0)	0 (0)
<1 month	0 (0)	1 (1.6)	1 (0.8)
1‐12 months	39 (61.9)	40 (63.5)	79 (62.7)
>1 year	24 (38.1)	22 (34.9)	46 (36.5)
Highest income earned in 1 year? (US $), n (%)			
≤26,999	3 (4.8)	4 (6.3)	7 (5.6)
27,000-35,999	9 (14.3)	10 (15.9)	19 (15.1)
36,000-51,999	24 (38.1)	28 (44.4)	52 (41.3)
52,000-69,999	12 (19.0)	12 (19.0)	24 (19.0)
70,000-99,999	15 (23.8)	9 (14.3)	24 (19.0)
≥100,000	0 (0)	0 (0)	0 (0)
PROMIS^a^ Depression at baseline, observed T-score, mean (SD)	55.9 (10.6)	56.9 (10.1)	56.4 (10.3)

a PROMIS: Patient-Reported Outcomes Measurement Information System.

**Figure 1.**
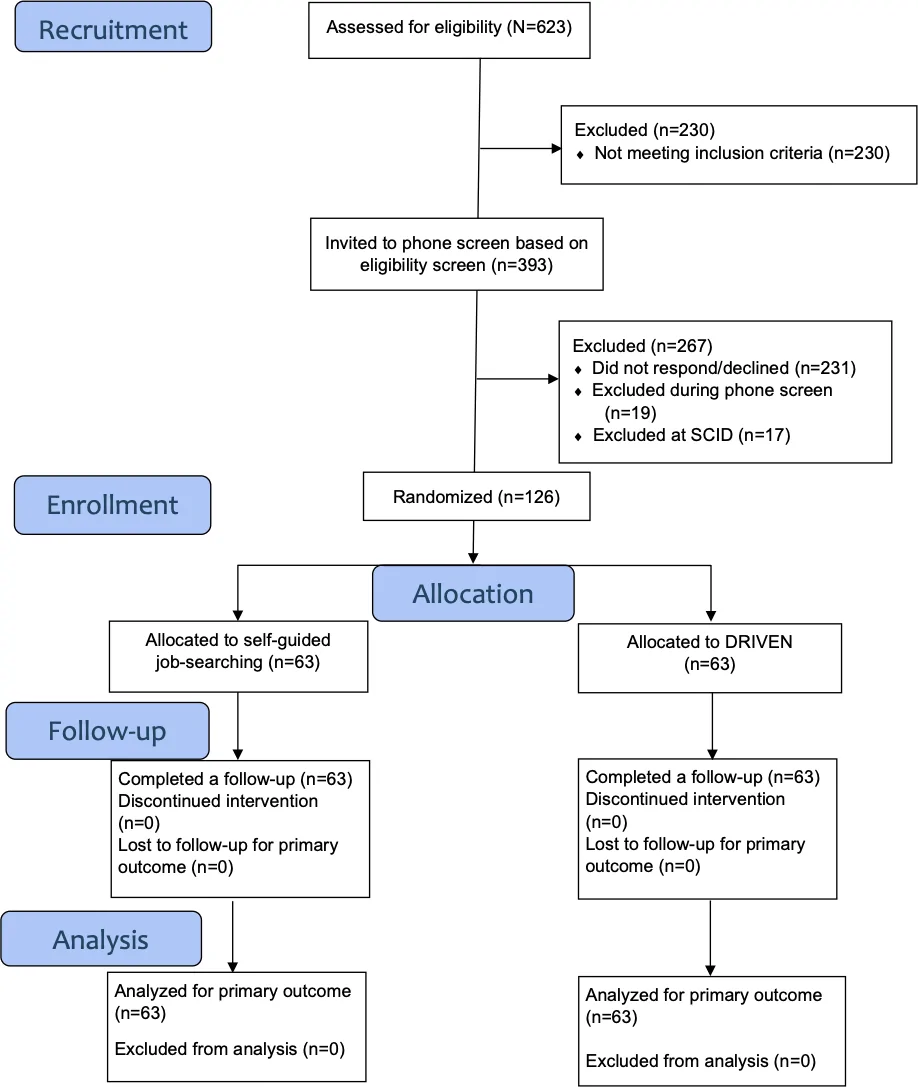
CONSORT flow diagram for DRIVEN RCT. CONSORT: Consolidated Standards of Reporting Trials; DRIVEN: Distress-Informed Return-to-Work Intervention; RCT: randomized controlled trial; SCID: Structured Clinical Interview for *DSM-5*.

**Table 3.** Estimated marginal means from mixed-effects regression models.

Time point and group	Job search: preparatory	Job search: active	Depression	Positive affect
Baseline and 6 weeks (covariate: baseline)				
Baseline				
Control, mean (SE)	14.1 (0.5)	11.8 (0.5)	56.9 (1.0)	41.8 (1.3)
DRIVEN^a^, mean (SE)	13.4 (0.5)	11.3 (0.5)	59.0 (1.0)	38.5 (1.3)
Standardized mean difference^b^ (95% CI)	–0.3 (–0.8 to 0.2)	–0.2 (–0.8 to 0.4)	0.4 (–0.2 to 1.0)	–0.6 (–1.3 to 0.1)
6 weeks				
Control, mean (SE)	14.6 (0.4)	12.8 (0.5)	51.8 (0.9)	50.3 (1.1)
DRIVEN, mean (SE)	15.6 (0.4)	13.8 (0.5)	50.0 (0.9)	51.4 (1.1)
Standardized mean difference (95% CI)	0.4 (0 to 0.8)^c^	0.4 (–0.1 to 0.9)	–0.4 (–0.9 to 0.1)	0.2 (–0.4 to 0.8)
Follow-up: 8 and 16 weeks, postintervention (covariate: 6 weeks)				
8 weeks				
Control, mean (SE)	16.1 (0.5)	15.4 (0.6)	49.4 (0.9)	50.3 (1.1)
DRIVEN, mean (SE)	15.9 (0.5)	14.5 (0.6)	50.6 (1.0)	51.5 (1.1)
Standardized mean difference (95% CI)	0 (–0.6 to 0.5)	–0.3 (–0.8 to 0.2)	0.2 (–0.3 to 0.7)	0.2 (–0.5 to 0.5)
16 weeks				
Control, mean (SE)	16.8 (0.5)	15.6 (0.5)	49.6 (0.9)	51.7 (1.0)
DRIVEN, mean (SE)	15.9 (0.5)	14.5 (0.5)	49.6 (0.9)	51.4 (1.1)
Standardized mean difference (95% CI)	–0.3 (–0.8 to 0.2)	–0.3 (–0.8 to 0.1)	0 (–0.4 to 0.5)	0 (–0.8 to 0.3)

a DRIVEN: Distress-Informed Return-to-Work Intervention.

b Standardized mean differences are calculated as DRIVEN – Control (ie, positive values favor DRIVEN).

c 95% CI contains zero (not visible due to rounding).

### Treatment Expectations

At the start of the trial, participants were asked to rate the credibility and expectations of the intervention to which they were randomized. For intervention credibility, DRIVEN participants had a mean of 22.5 (SD 4.0), while control participants had a mean of 23.7 (SD 3.6); 2-tailed Welch *t*_122.77_=1.82; *P*=.07. For expectations of change, DRIVEN participants had a mean of 15.2 (SD 3.1), while control participants had a mean of 15.5 (SD 3.0); Welch *t*_123.18_=0.66; *P*=.51. In sum, there were no differences in expectations or credibility between the groups.

### Efficacy of the DRIVEN Intervention: Job-Related Outcomes

The rate of missing data was low in this sample, with less than 5% at each time point for the primary outcomes. In a test of our primary hypothesis, we found that participants receiving the DRIVEN intervention exhibited significantly more job-search behavior. In the DRIVEN group, preparatory job-search behavior increased significantly by 0.053 (95% CI 0.031-0.076) units per day since baseline. In the control condition, the increase in job-search behavior was smaller (0.01-unit increase for each day, 95% CI –0.011 to 0.033; not significant). Importantly, the contrast of these 2 quantities was statistically significant: Welch *t*_604_=2.658; *P*=.008. A test of whether *time* (continuous) had a nonlinear trajectory over the course of the study was not significant, as inclusion of a *time-squared* effect did not significantly improve the model (likelihood ratio test=0.11; *P*=.95). Results of this hypothesis are shown in Figure 2. Importantly, there was a high level of individual differences across trajectories over time, suggesting caution in interpreting summary statistics (Figure 2A).

**Figure 2.**
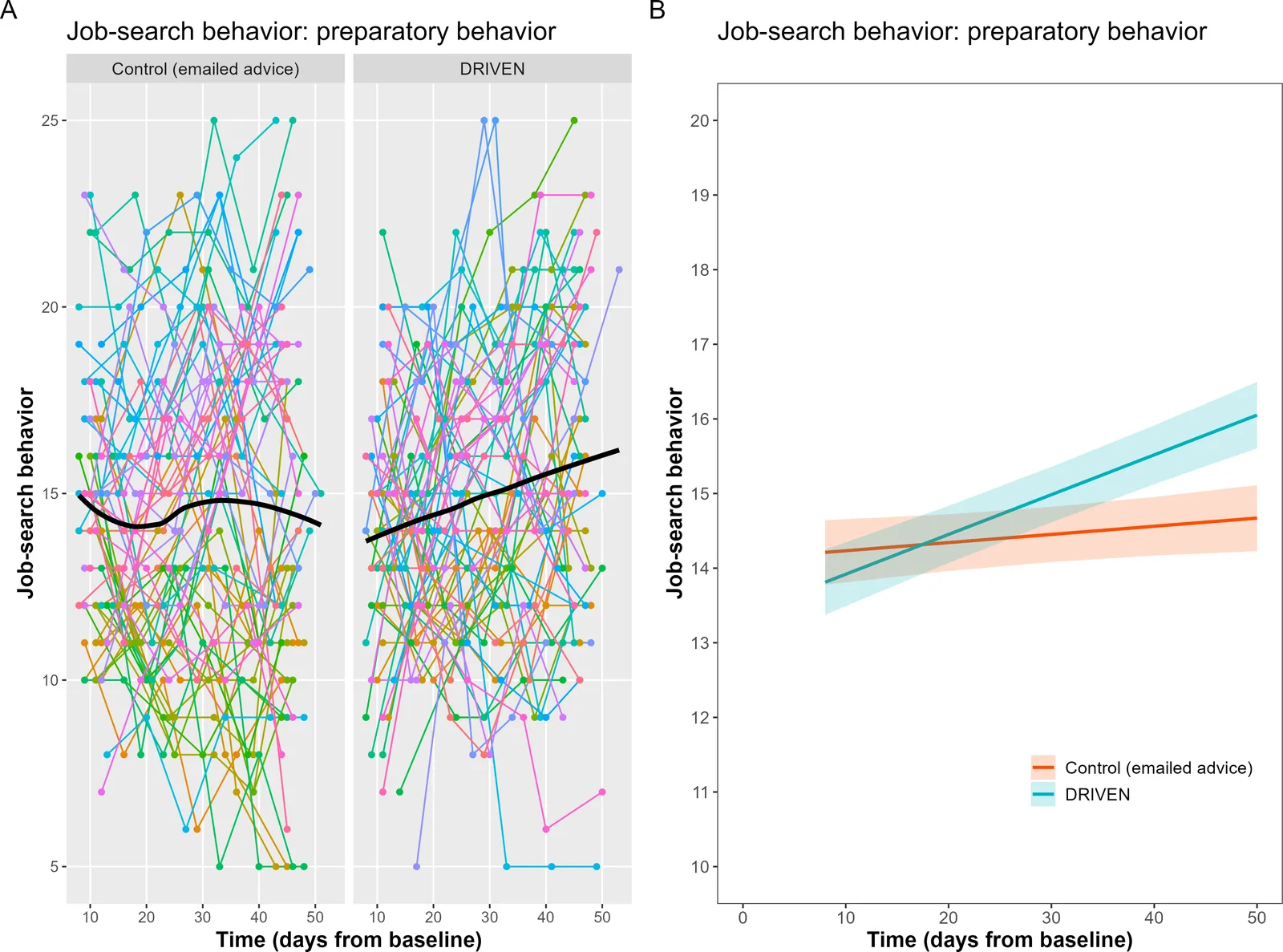
Differences between the DRIVEN and control groups with respect to the preparatory behavior subscale of the Job Search Behavior Scale. (A) Trajectories of individual participants are shown, overlaid with a LOESS line, which indicates an approximately linear trajectory of job-search behavior over time. (B) The fitted linear trajectories from the linear mixed model are shown with ±1 SE, indicating that the DRIVEN intervention is associated with more preparatory job-search behavior over time. DRIVEN: Distress-Informed Return-to-Work Intervention; LOESS: locally estimated scatterplot smoothing.

For active job-search behavior, a similar pattern was evident (Figure 3). The increase in active job-search behavior for those in DRIVEN was 0.061 (95% CI 0.038-0.084), whereas the control group experienced a smaller increase over time in active job search (point estimate: 0.025, 95% CI 0.001-0.047); Welch *t*_608_=2.226; *P*=.03. Collectively, Figures 2 and 3 show the difference in trajectory between the DRIVEN and control groups in terms of job seeking, as well as a high degree of person-to-person variability.

**Figure 3.**
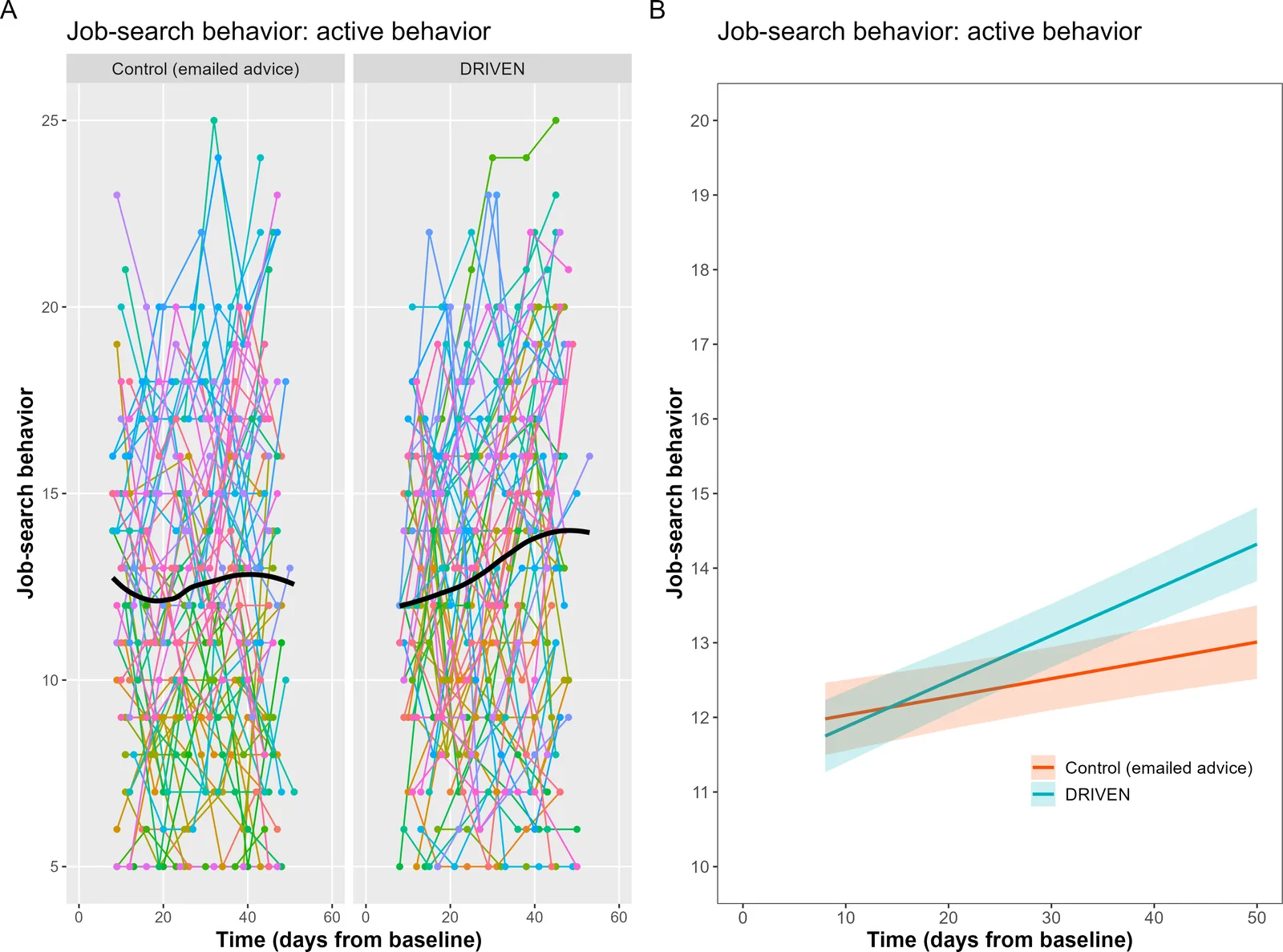
Differences between the DRIVEN and control groups with respect to the active behavior subscale of the Job Search Behavior Scale. (A) Trajectories of individual participants are shown, overlaid with a LOESS line, which indicates an approximately linear trajectory of active job-search behavior over time. (B) The fitted linear trajectories from the linear mixed model are shown with ±1 SE, indicating that the DRIVEN intervention is associated with more active job-search behavior over time. DRIVEN: Distress-Informed Return-to-Work Intervention; LOESS: locally estimated scatterplot smoothing.

As shown in Table 3, the groups were neither statistically nor substantively different at the 8- and 16-week follow-ups on the 2 job-search measures. However, the groups differed on our secondary outcome of tangible job-attainment. In the DRIVEN group, 68.3% (43/63) of participants found a job compared with 50.8% (32/63) in the control group, a statistically significant and meaningful difference (*χ*_1_^2^=4.0; *P*=.046). The groups did not differ on time to job-finding (also a secondary outcome), with the difference between the groups emerging at the last time point of the study (Supplemental Results and Figure S1 in Multimedia Appendix 1).

### Efficacy of the DRIVEN Intervention: Secondary Outcomes on Depression and Positive Affect

We also examined differences in change over time (measured using continuous time since baseline) between the 2 groups on PROMIS Depression T-scores. The DRIVEN group showed a decrease in depression (point estimate=–0.214, 95% CI –0.255 to –0.172) over the course of the study, a decrease that was significantly larger than that observed in the control group (point estimate=–0.122, 95% CI –0.163 to –0.081); Welch *t*_604_=3.113; *P*=.002.

A similar pattern was observed for PROMIS positive affect T-scores. The DRIVEN group showed an increase in positive affect (point estimate=0.306, 95% CI 0.260-0.352) over the course of the study, an increase that was significantly larger than that observed in the control group (point estimate=0.202, 95% CI 0.156-0.248); Welch *t*_604_=3.132; *P*=.002.

## Discussion

### Summary of Main Findings

We found that a 6-week app-based intervention that integrated teaching job-search behaviors and distress-reduction strategies was efficacious for (1) increasing job-search behavior, (2) increasing the odds of finding a job during the study period, and (3) reducing depression. These effects were statistically significant, even relative to an active control group in which people received standard job-seeking advice by email. We observed small-to-medium-sized effects, and these were not sustained in the 8- and 16-week postintervention periods, where the 2 groups no longer differed in job-search behavior, depression, or positive affect.

### Implications

These results are relevant to psychiatric care. Mental disorders often co-occur with, and can maintain, joblessness [26], but there are few scalable treatments that focus specifically on helping job seekers return to the workforce that also take their mental health into account. Pharmacotherapy and behavioral interventions that are commonly used for depression and anxiety focus on treating symptoms, which in turn are expected to help improve functional domains such as being able to attain and keep a job. Although interventions exist for unemployment and the intersection with mental health, they are not in wide use and mixed findings on their efficacy have led to calls for more research [27]. Moreover, a recent Cochrane review and meta-analysis found that the few employment interventions that included “health-improving” components (eg, CBT and exercise) improved employment rates, but had mixed results, preventing broad and definitive conclusions about employment interventions due to low-quality evidence [28].

DRIVEN, therefore, fills a public health need as it is scalable, being largely delivered via a smartphone app, including 3 sessions with a job coach to support engagement with the intervention as well as the participant’s job search. Moreover, engagement with the DRIVEN app was high (>84%; Table S2 in Multimedia Appendix 1), suggesting a high degree of acceptability. However, more research is needed on the durability of the intervention, as the effects were no longer present during follow-up periods. In addition, given the heterogeneity of the needs of job seekers, it is likely that a “precision medicine” approach may be needed [29], where more attention and resources are allocated to higher-need participants, and specific techniques are matched to the needs of the job seeker.

To increase access to interventions for unemployed job seekers, interventions such as DRIVEN can be delivered in venues beyond traditional mental health clinics. First, federal, state, and local government agencies typically offer basic career training, job fairs, online resources, and resume guidance [30]; app-based interventions like DRIVEN can be easily implemented in these contexts. Second, private companies sometimes offer outplacement services prior to employee dismissal (eg, career counseling resources to support transitions to a new job) and are often interested in addressing the mental health needs of their recently laid-off employees, suggesting another venue for DRIVEN [31]. Partnering with these organizations could expand the possible mental health and economic impact of interventions like DRIVEN.

### Future Directions

This is the first RCT of DRIVEN, a cognitive behavioral intervention designed for job seekers that addresses the emotional and practical needs of unemployed individuals. Subsequent work should seek to replicate these findings, as well as to determine the generalizability to other populations (eg, in a mental health setting where all participants have a mental health diagnosis). The variability in change over time suggests that tailoring the intervention may be needed (eg, certain individuals may benefit from a more intensive intervention or more coaching sessions). Modifications to the program may be needed for specific subpopulations, as well as for underemployed individuals seeking to improve work-related occupational functioning.

This study focuses on whether DRIVEN works, and subsequent analyses will focus on the mechanisms of change for this intervention (ie, why it works). In our protocol, we proposed to analyze which changes in psychological constructs lead to changes in job-seeking behavior, and vice versa. We intend to publish these results in the future as a separate journal article. The hypothesized mechanisms of DRIVEN focus on *thinking flexibly* and *taking action*, two aspects of CBT. Other employment-related interventions focus on additional mechanisms of change, such as interpersonal competence and the reduction of social anxiety [32]. Specific mechanisms of job-support interventions remain an important direction for future studies.

### Limitations

Although these initial results are encouraging, some limitations must be noted. First, participants were recruited online from major US metropolitan areas and completed the intervention remotely. Although this approach is advantageous for increasing access, it may not generalize well to certain locales (eg, rural areas) and other health care settings (eg, a medical center environment). In addition, the use of Craigslist for recruitment limits generalizability to the broad population of job seekers. Second, we excluded people with suicidal ideation. This was done for reasons of participant safety. However, the association between suicidality and unemployment is well documented [33]. Third, there were limitations in scope; the DRIVEN program was only available in English, and only available to those with access to a smartphone. Although this represents a large population, we hope to remedy this in future iterations of the program. Fourth, in trials of behavioral interventions, it is difficult to maintain blinding because patients are aware of the procedures in their received intervention (for a review of issues related to blinding in behavioral intervention trials, Juul et al [34]). Study staff were not blind to assigned treatment group because research team members needed to set up participants for the study based on their assigned condition. That said, knowledge of the active vs control group was masked from participants, and the 2 groups did not differ in terms of expectations. Fifth, participants in both groups of this study were paid for participation and could earn up to US $350 over the course of the study for completing study assessments. In real-world implementations of interventions, participants are typically not paid. Thus, whether DRIVEN is effective in future implementations of the program remains to be seen and will be the subject of future studies. Finally, groups did not significantly differ on self-reported outcomes during the follow-up periods, or on time taken to find a job. Future studies may need to consider a slightly longer intervention, especially for people who have been unemployed for a long time and need intensive support.

### Summary and Conclusions

In sum, compared with a control condition designed to reflect job seeking as usual, a scalable, smartphone-based intervention that integrates mental health with job-seeking strategies (DRIVEN) was more efficacious in improving job-search behaviors, reducing depression, and enhancing positive affect from baseline to the 6-week follow-up, ultimately increasing the likelihood of obtaining a job. Given the robust impact of unemployment on mental health across mental disorders [35], these results further point to the importance of targeting employment status as a public health outcome. As rates of depression may be increasing [36], combined with economic uncertainties, interventions that acknowledge the intersection of employment and mental health may be increasingly needed.

## Supplementary material

10.2196/90873 Multimedia Appendix 1 Additional details on the inclusion and exclusion criteria, harms assessment, engagement with DRIVEN exercises, supplemental analyses and figures, and screenshots of the mobile app.

10.2196/90873 Checklist 1 CONSORT 2025 checklist.

## Abbreviations

CBT: cognitive behavioral therapy
CONSORT: Consolidated Standards of Reporting Trials
DRIVEN: Distress-Informed Return-to-Work Intervention
PROMIS: Patient-Reported Outcomes Measurement Information System
RCT: randomized controlled trial
