# Supplementary material for: Job Coaching and Smartphone-Based Cognitive Behavioral Techniques for Returning to Work and Coping With Unemployment (DRIVEN): Randomized Controlled Trial
Source: J Med Internet Res. 2026 Oct 20;28:e90873. doi: 10.2196/90873 (PMC13717267; doi:10.2196/90873)
Supplement: Supplementary file 1 [file jmir-v28-e90873-s001.docx]

**ONLINE SUPPLEMENT FOR:**

**Job Coaching and Smartphone-Based Cognitive Behavioral Techniques for Returning to Work and Coping With Unemployment (DRIVEN): Randomized Controlled Trial**

| **Table S1. Inclusion and Exclusion criteria** | |
| --- | --- |
| **Inclusion Criteria** | **Exclusion Criteria** |
| - 18-65 years of age - Currently unemployed (working <8 hours per week) & actively searching for work - Prior to unemployment, were employed full-time (35 hours per week or more) for at least 1 year - Prior to unemployment, received an individual highest annual salary of $100,000 or less - Owns an iPhone or Android Phone - Willing and able to provide informed consent - Has a minimum depression T score of 45 according to the PROMIS 8A Depression scale - Able to communicate in English (verbal and written) | - Current moderate or severe alcohol or substance use disorders - Current manic episode or psychotic symptoms - Currently employed, retired or a student (part-time or full-time) - Current suicidal plan and/or active suicidal ideation |

**Assessment of Harms**

We did not have any hypotheses about possible harms from the intervention. The possibility of psychological harm was assessed using the PROMIS Depression scale, for the possibility that the intervention might increase depression, rather than decrease depression.

**Supplemental Results**

**Table S2**

**Engagement with mobile app exercises by three types of activity**

**Supplemental Results**

**Survival analyses**

The survdiff() function was used to compare survival curves for the DRIVEN versus the control group, with all participants (*N* = 126) included in the analysis. No significant difference was found between the survival curves (Χ^2^ = 1.6, df = 1, *p* = .2). As shown in Figure S1, there were small differences between the groups’ trajectories up until the last time point of the study, at which time an advantage was observed for the DRIVEN group.

**FIGURE S1. SURVIVAL ANALYSES OF JOB FINDING.** The survival curves did not statistically significantly differ, although an advantage for the DRIVEN group is visible at the end of the study (right side of x axis).

**Supplemental Material: Screenshots of DRIVEN Program**

**Sample Navigation and Welcome Screens.**

**Sample Exercise Screens.**

**Sample Exercise and Instructional Video Screens**
